# Therapeutic interventions aimed at cccDNA: unveiling mechanisms and evaluating the potency of natural products

**DOI:** 10.3389/fcimb.2025.1598872

**Published:** 2025-06-17

**Authors:** Liyuan Hao, Shenghao Li, Xiaoyu Hu

**Affiliations:** ^1^ School of Clinical Medicine, Chengdu University of Traditional Chinese Medicine, Chengdu, Sichuan, China; ^2^ Department of Infectious Diseases, Hospital of Chengdu University of Traditional Chinese Medicine, Chengdu, Sichuan, China

**Keywords:** cccDNA, natural product, HBV, covalently closed circular DNA, hepatitis B virus

## Abstract

Hepatitis B virus (HBV) infection persists as a formidable global health predicament, imposing a substantial burden on public health. It not only elevates the risk of cirrhosis but also significantly heightens the incidence of hepatocellular carcinoma (HCC), thereby exacerbating the complexity of managing this disease. Central to the intractability of chronic hepatitis B is the tenacious persistence of covalently closed circular DNA (cccDNA) within the nuclei of infected hepatocytes. This cccDNA serves as a stable transcriptional template, continuously fueling the production of viral components and rendering the virus refractory to current antiviral interventions.​ The attainment of a definitive cure for HBV infection hinges upon the development of innovative antiviral strategies that can precisely and effectively target and eliminate cccDNA from the infected liver cells. In this regard, natural products have emerged as a promising source of potential therapeutics. This comprehensive review delves into the natural products that have shown promise in specifically targeting cccDNA. It meticulously elucidates the intricate molecular mechanisms through which these natural compounds modulate cccDNA activity, such as interfering with cccDNA formation, disrupting its epigenetic regulation, or inhibiting its transcriptional output. Developing innovative strategies to target and eliminate cccDNA is crucial for curing HBV infection, and natural products hold great promise. This review details several natural products with cccDNA-targeting potential, supported by clear mechanisms and data. Dehydrocheilanthifolin (DHCH) from *Corydalis saxicola* inhibits HBsAg and HBeAg secretion in HepG2.2.15 cells. It may disrupt viral processes like pgRNA packaging or DNA polymerase activity, with IC50 values for reducing extracellular, intracellular DNA, and cccDNA at 15.08 μM, 7.62 μM, and 8.25 μM respectively. Methyl helicterate from *Helicteres angustifolia* decreases HBsAg, HBeAg, HBV DNA, and cccDNA in HepG2.2.15 cells. 15.8 μM reduces intracellular cccDNA. Curcumin from turmeric reduces viral load and cccDNA in d-imHCs; 30µM halves cccDNA levels. Epigallocatechin gallate (EGCG) from green tea hinders viral transcription and replication. 22.9μg/ml EGCG lowers cccDNA by about 60%. Asiaticoside from *Hydrocotyle sibthorpioides* inhibits HBsAg, HBeAg, and cccDNA in HepG2.2.15 cells. Notably, despite extensive research, no natural product has yet obtained clinical validation for cccDNA clearance, highlighting the significant translational gap between pre-clinical research and clinical application. By elucidating these molecular mechanisms, this review aims to contribute to the development of HBV-targeted therapies, offering valuable insights for designing novel therapeutic agents and optimizing existing treatment regimens, ultimately advancing the quest for an effective cure for HBV infection.

## Graphical Abstract

After HBV infects hepatocytes, its genome forms cccDNA in the nucleus, resulting in chronic infection (CHB). Natural products can control the progression of diseases by inhibiting the formation of cccDNA.

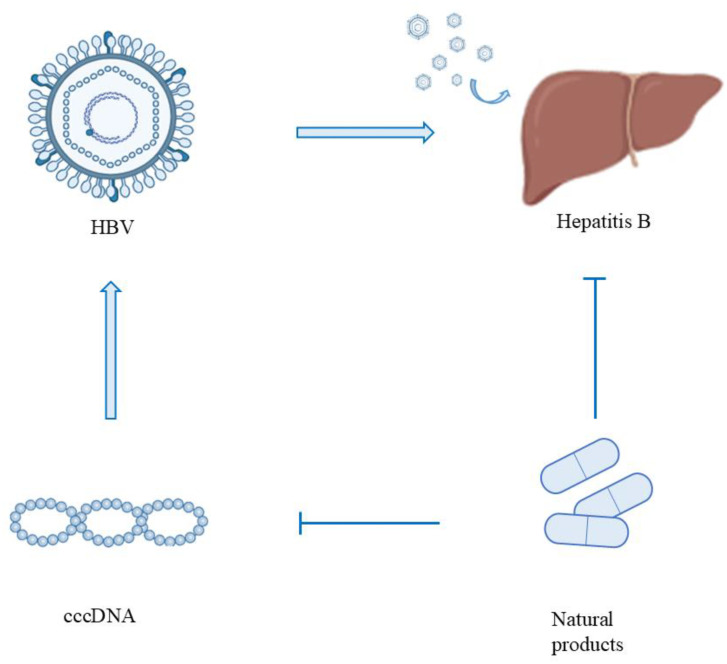

## 1 Introduction

**Figure 1:**
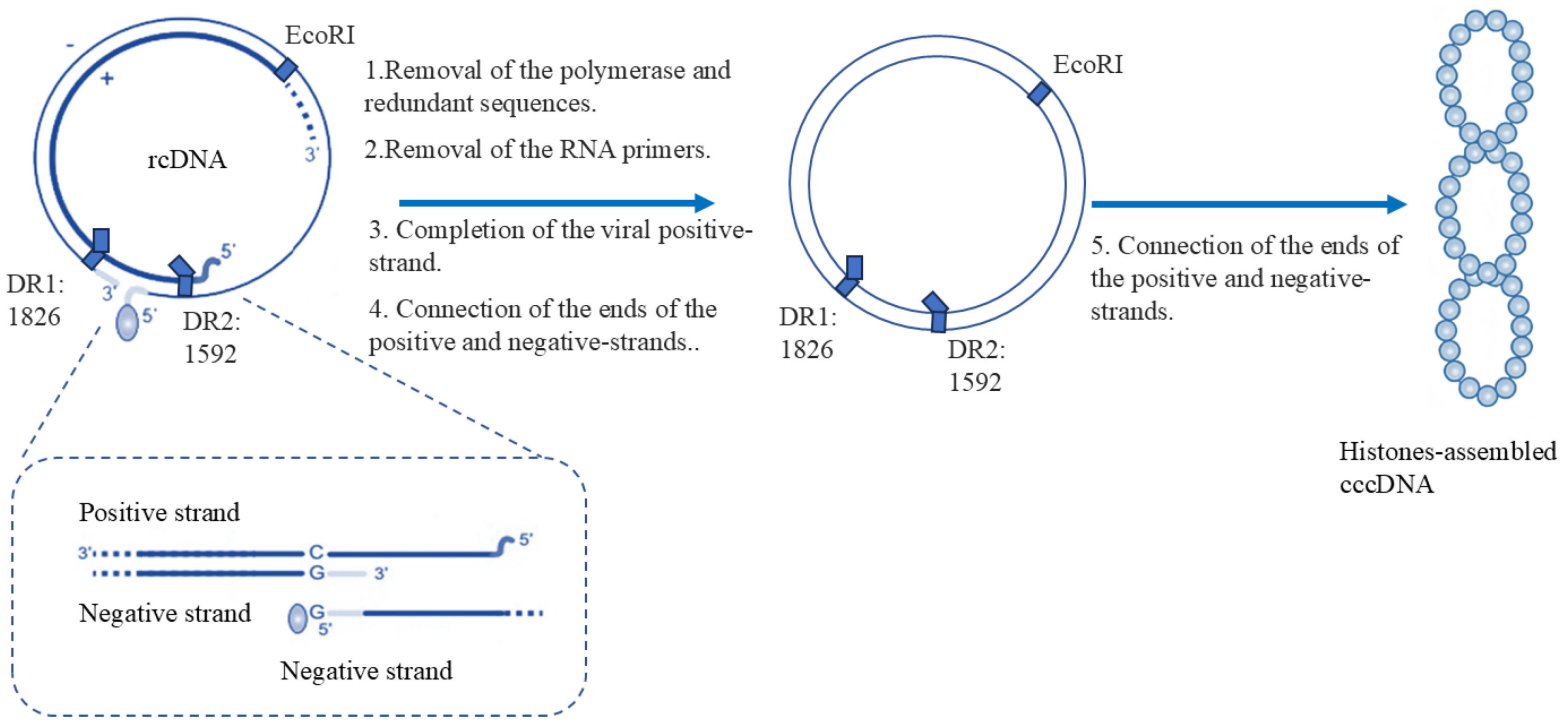
Mechanisms and structural differences in rcDNA-to-cccDNA transformation. The conversion of rcDNA to cccDNA involves the following sequential steps:(1) The viral polymerase attached to the 5’-end of the negative strand is removed, resulting in protein-free rcDNA (pf-rcDNA). (2) The RNA primer at the 5’-end of the positive strand is removed. (3) Using the negative strand as a template, the positive strand of viral DNA is completed. (4) The ends of both the positive and negative strands are ligated. (5) The DNA undergoes hyper - helical coiling and associates with histones to form the cccDNA minichromosome. cccDNA consists of two fully formed strands: the minus - strand on the outer part and the plus-strand on the inner part. At nucleotide (nt) positions 1826 and 1592, there are two direct repeats (DRs). Moreover, the location of the origin is at the EcoRI site.

Hepatitis B virus (HBV) is one of the most common human pathogens responsible for both acute and chronic hepatitis. Globally, an estimated 257.5 million people were infected with HBV in 2022, with a global HBV prevalence of 3.2%. The global diagnosis and treatment rates for hepatitis B are 14% and 8%, respectively. The global number of hepatitis B related deaths is expected to increase from 858,000 in 2015 to 1.149 million in 2030. During the same period, the number of liver cancer cases is projected to rise from 644,000 to 857,000, and the number of decompensated cirrhosis cases is anticipated to increase from 296,000 to 403,000 (Global prevalence, cascade of care, and prophylaxis coverage of hepatitis B in 2022: A modelling study, 2023). The Diagram of HBV Infection is shown in **Figure 1**.

HBV is an enveloped, non-cytolytic virus belonging to the *Hepadnaviridae* family (Seeger and Mason, 2015). Its genome is a compact, circular, partially double-stranded DNA (3.2 kb) enclosed within a nucleocapsid core and surrounded by an outer lipoprotein layer. The HBV genome contains four overlapping open reading frames (ORFs) (Van Damme et al., 2021). The infection process begins with a low-affinity interaction between viral envelope proteins and heparan sulfate proteoglycan (HSPG), followed by a high-affinity binding to its receptor, sodium taurocholate co-transporting polypeptide (NTCP), which facilitates viral entry (Yan et al., 2012; Verrier et al., 2016). Additionally, the epidermal growth factor receptor (EGFR) facilitates the internalization of HBV into hepatocytes. Once inside the cytoplasm, the virus transports its nucleocapsid into the nucleus, where the viral DNA converts from a relaxed circular form (rcDNA) to covalently closed circular DNA (cccDNA) (Iwamoto et al., 2019; Tsukuda and Watashi, 2020). The cccDNA serves as a template for the production of pregenomic RNA (pgRNA) and various subgenomic RNAs. pgRNA is packaged into the nucleocapsid, where it undergoes reverse-transcription to form the DNA negative strand. The negative strand then serves as a template for the synthesis of the positive strand, resulting in the formation of rcDNA as the major form and dslDNA as the minor form. The rcDNA can be returned to the nucleus and recycled to augment the cccDNA pool. In addition, the nucleocapsid needs to be transported from its location within the cell to the vicinity of the cell membrane. The envelope of HBV consists of three related proteins, called large (L), medium (M), and small (S) hepatitis B surface antigens (HBsAg) (Harvey et al., 1999). The S protein drives the secretion of subviral particles subviral particles (SVPs) (Bi and Tong, 2018). SVPs are particle structures that do not contain the viral genome but play an important role in the interaction between viral infection and the host immune response. Research has shown that SVPs may be a strategic mechanism used by viruses to evade host immune responses (Vaillant, 2021). It has been found that SVPs can interfere with the recognition process of immune cells or induce immune tolerance in immune cells, thus helping the virus escape the attack of the immune system (Selim et al., 2024). L-HBsAg is believed to mediate contact between the virus envelope and nucleocapsid protein (HBcAg) (Tan et al., 1999). In the assembly of infectious virions, the pre-S and S regions of L-HBsAg interact synergistically with hepatitis B core Antigen (HBcAg) (Muhamad et al., 2015).

Currently, the primary therapeutic options for HBV are pegylated-interferon-α (PEG-IFN-α) and nucleos(t)ide analogues (NAs) (Pan et al., 2023). Although these drugs can effectively inhibit HBV replication, reduce inflammation, and improve the prognosis of patients, they are unable to eliminate the initial cccDNA formed in the nuclei of infected liver cells. Consequently, patients typically need to undergo long-term antiviral therapy to suppress viral replication (Martinez et al., 2021; Korkmaz et al., 2023). It is essential to clarify the difference between sterilizing cure and functional cure. A sterilizing cure refers to the complete eradication of HBV, including the elimination of cccDNA from infected hepatocytes, offering the potential for a permanent resolution of the infection. In contrast, a functional cure aims to control the virus to the extent that patients no longer experience disease progression, despite the possible persistence of low levels of cccDNA. Therapeutic strategies targeting cccDNA are crucial for both outcomes. For a functional cure, interfering with cccDNA activity, such as inhibiting its transcription or disrupting its epigenetic regulation, can suppress viral replication to a level where the immune system can maintain control, reducing the risk of cirrhosis and hepatocellular carcinoma. For achieving a sterilizing cure, however, complete elimination of cccDNA is necessary, which requires the development of drugs capable of degrading or removing cccDNA from the nucleus. Therefore, there is an urgent and crucial need to develop effective anti-cccDNA drugs and identify novel targets and methods to disrupt the HBV infection and replication processes, aiming to move closer to either a functional or sterilizing cure for HBV infection.

## 2 Methods

Literature Search Strategy: A comprehensive literature search was conducted across three authoritative scientific databases: PubMed, Embase, and Web of Science. A set of carefully selected keywords and MeSH terms was utilized to comprehensively cover the research domain. The search terms included “HBV”, “cccDNA”, “Natural products”, “Anti-HBV therapy”, “HBV replication”, “HBV transcription”, “HBx protein”, “HBeAg”, “HBsAg”, “HBc protein”, etc. Boolean operators (AND, OR) were employed to combine these keywords, optimizing the search precision and recall. Our literature search covered all relevant studies from the establishment of the databases to before January 1, 2025.

Inclusion and Exclusion Criteria: Inclusion criteria: Studies were included only if they investigated the effects of natural products on HBV cccDNA, covering aspects such as cccDNA formation, transcription, and stability. Only original research articles based on experimental studies, including cell-based experiments, animal models, or clinical trials, were eligible. Articles had to be published in English. Exclusion criteria: comments, conferences, editorials, letters and replies were excluded. Studies unrelated to HBV cccDNA or natural products were omitted. In cases of duplicate publications, only the article with the most comprehensive.

## 3 Overview of cccDNA

cccDNA is formed from rcDNA delivered by viral particles and serves as the sole template for viral pgRNA and all subgenomic mRNAs. The conversion of rcDNA to cccDNA involves the following sequential steps: (1) The viral polymerase attached to the 5’-end of the negative strand is removed, resulting in protein-free rcDNA (pf-rcDNA). (2) The RNA primer at the 5’-end of the positive strand is removed. (3) Using the negative strand as a template, the synthesis of the positive strand of viral DNA is completed. (4) The ends of both the positive and negative strands are ligated. (5) The DNA undergoes hyper-helical coiling and associates with histones to form the cccDNA minichromosome (**Figure 1**) (Zhu et al., 2019). The cccDNA molecule contains four open reading frames (ORFs): surface (S), pre-core/core (C), polymerase (P), and X, which encode seven proteins: HBc (the viral capsid protein), hepatitis B virus polymerase/reverse transcriptase (HBV POL/RT) L, M and S proteins (preS1, preS2 and S domains, which are envelope proteins), and hepatitis B x antigen (HBx, a transcriptional activator). After the pgRNA and polymerase are encapsulated within the nucleocapsids, the pgRNA is reverse transcribed into rcDNA by the viral polymerase (Martinez et al., 2022). Subsequently, a portion of the nucleocapsids are packaged into new infectious particles (Dane particles), while another portion is transported back to the nucleus to replenish the cccDNA pool (Martinez et al., 2022). Consequently, cccDNA is critical for HBV persistence, and even a few copies can sustain infection after treatment cessation (Fanning et al., 2019).

## 4 Transcriptional regulation mechanism of cccDNA

**Figure 2:**
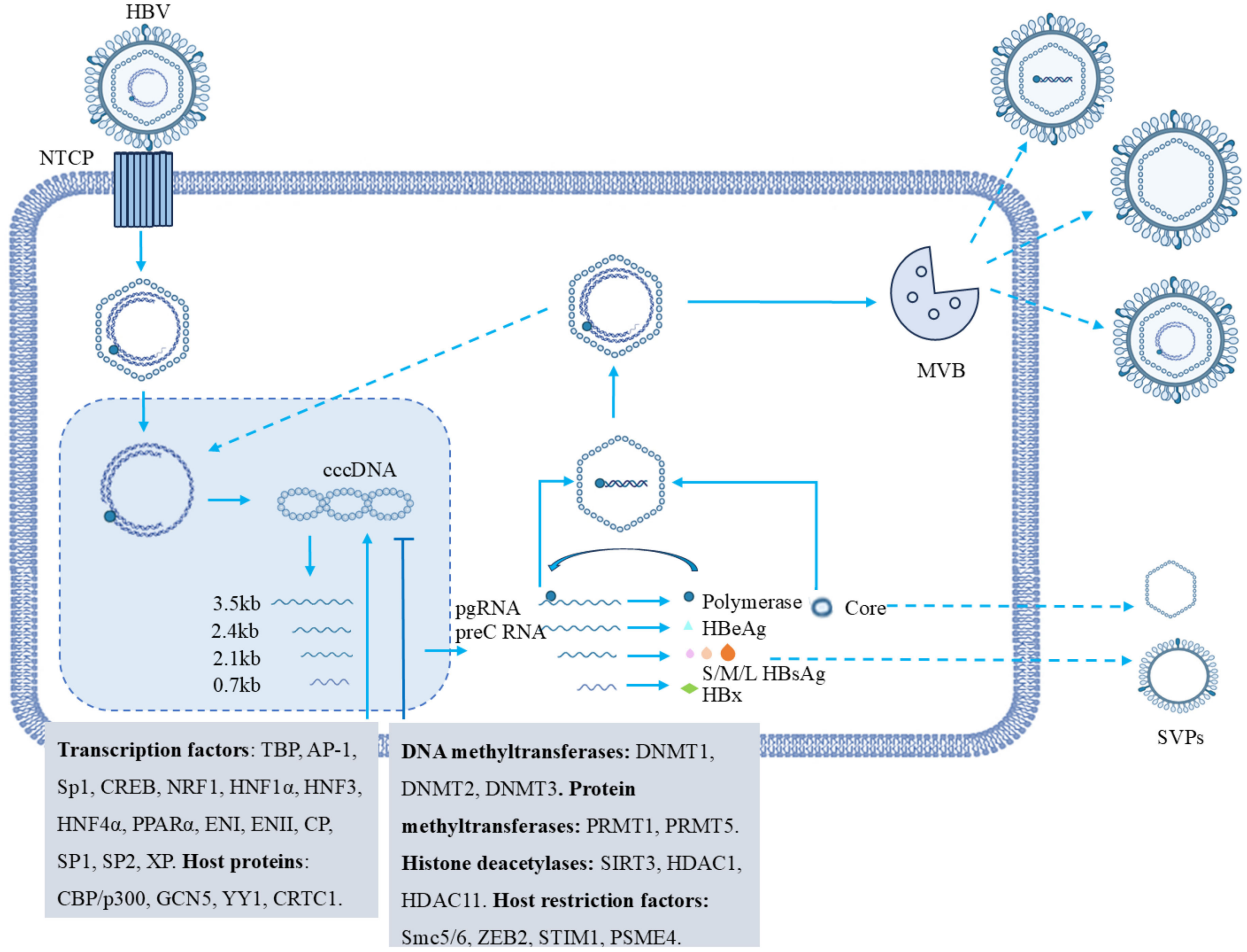
Regulatory factors of cccDNA transcription in HBV. The HBV life cycle begins with HBV binding to the NTCP receptor on hepatocytes. The viral nucleocapsid then enters the nucleus, where rcDNA is converted to cccDNA. Using cccDNA as a template, transcription generates different RNAs that synthesize specific viral proteins. PgRNA interacts with polymerase, gets packaged into capsids, and through reverse transcription, a complete viral genome is formed. Post-replication, HBV particles and non-infectious SVPs are assembled and secreted, potentially with multivesicular bodies (MVBs) involved in transport. Various host and viral factors that are involved in cccDNA regulation. These include transcription factors (e.g., TBP, AP-1, Sp1), DNA methyltransferases (DNMT1, DNMT2, DNMT3), protein methyltransferases (PRMT1, PRMT5), histone deacetylases (SIRT3, HDAC1, HDAC11), and host restriction factors (Smc5/6, ZEB2, STIM1, PSME4).

The transcription of cccDNA is a crucial step in the HBV life cycle. This process is regulated by both host factors and viral proteins (**Figure 2**). Functional cure of hepatitis B is defined as the persistent undetectability of circulating HBsAg and HBV DNA after a finite course of treatment (Wong et al., 2022). Targeting cccDNA transcription is an important means to improve the functional cure rate of hepatitis B. Studies have demonstrated that numerous transcription factors bind to the enhancer or promoter region of the HBV genome, thereby regulating its transcription. These transcription factors include the TATA Box protein (TBP), activating protein 1 (AP-1), specific protein 1 (Sp1), cAMP response element-binding transcription factor (CREB), nuclear respiratory factor 1 (NRF1), and liver tissue-enriched transcription factors such as hepatocyte nuclear factor 1α (HNF1α), HNF3, HNF4α, peroxisome proliferator activated receptor α (PPARα), binding agents (including the two enhancers ENI and ENII), or promoter regions (including the genome containing a core promoter (CP), preS1 promoter (SP1), preS2 promoter (SP2), and X promoter (XP)) (Yoon et al., 2004; Qin et al., 2016; Turton et al., 2020).

Epigenetic modifications of cccDNA, such as DNA methylation and histone modification, play a role in regulating the transcriptional activity of cccDNA. Studies have shown that various host factors including CREB, CREB-binding protein (CBP)/p300, general control nonderepressible 5 (GCN5), Yin Yang 1 (YY1) and CREB-regulated transcriptional coactivator 1 (CRTC1), bind to cccDNA and promote its transcription (Hayashi et al., 2000; Belloni et al., 2009; Tang et al., 2014). Conversely, DNA methyltransferases (DNMT1, DNMT2, DNMT3) cause high methylation of cccDNA by acting on CpG islands in the HBV genome, inhibiting its transcriptional activity (Vivekanandan et al., 2010). In addition, multiple protein methyltransferases, such as protein arginine methyltransferase 1 (PRMT1) and PRMT5, have also been shown to reduce cccDNA transcription by regulating histone methylation levels of cccDNA (Benhenda et al., 2013; Zhang et al., 2017). Histone deacetylases sirtuin 3 (SIRT3), histone deacetylase 1 (HDAC1), HDAC11 reduce histone acetylation levels and inhibit transcriptional activity of cccDNA (Guo et al., 2011; Ren et al., 2018; Yuan et al., 2019). In recent years, with the advancement of high-resolution mass spectrometry, many novel histone modification types, such as crotonylation, succinylation, propionylation and malonylation, have been discovered (Kebede et al., 2017; Wang et al., 2017; Wei et al., 2017; Ishiguro et al., 2018). These findings provide a basis for further understanding of epigenetic modification types of cccDNA.

With the deepening understanding of the role of host factors in the regulation of cccDNA transcription, HBx plays a key role in the initiation and maintenance of cccDNA transcription. Parvulin 14 and Parvulin 17 bind to HBx and cccDNA and promote HBV replication in an HBX-dependent manner (Saeed et al., 2019). When HBx is absent, the histone methyltransferases SETDB1 and PRMT1 bind to cccDNA, causing the transcriptional activity of cccDNA to decrease (Rivière et al., 2015). Studies have shown that when HBx is present, PCAF/GCN5 and CBP/p300 are recruited to cccDNA, and the binding of hSirt1 and HDAC1 to cccDNA is reduced, activating the transcription of cccDNA (Belloni et al., 2009). In addition, HBx also induces host limiting factor Smc5/6, zinc finger E-box binding homeobox 2 (ZEB2), stromal interaction molecule 1 (STIM1) and proteasome activator subunit 4 (PSME4) degradation, enhancing cccDNA transcription activity (Decorsière et al., 2016; Minor et al., 2020). Therefore, HBx itself and HBx-involved protein-protein interactions are novel molecular targets for therapeutic development.

## 5 Natural products and cccDNA

**Figure 3:**
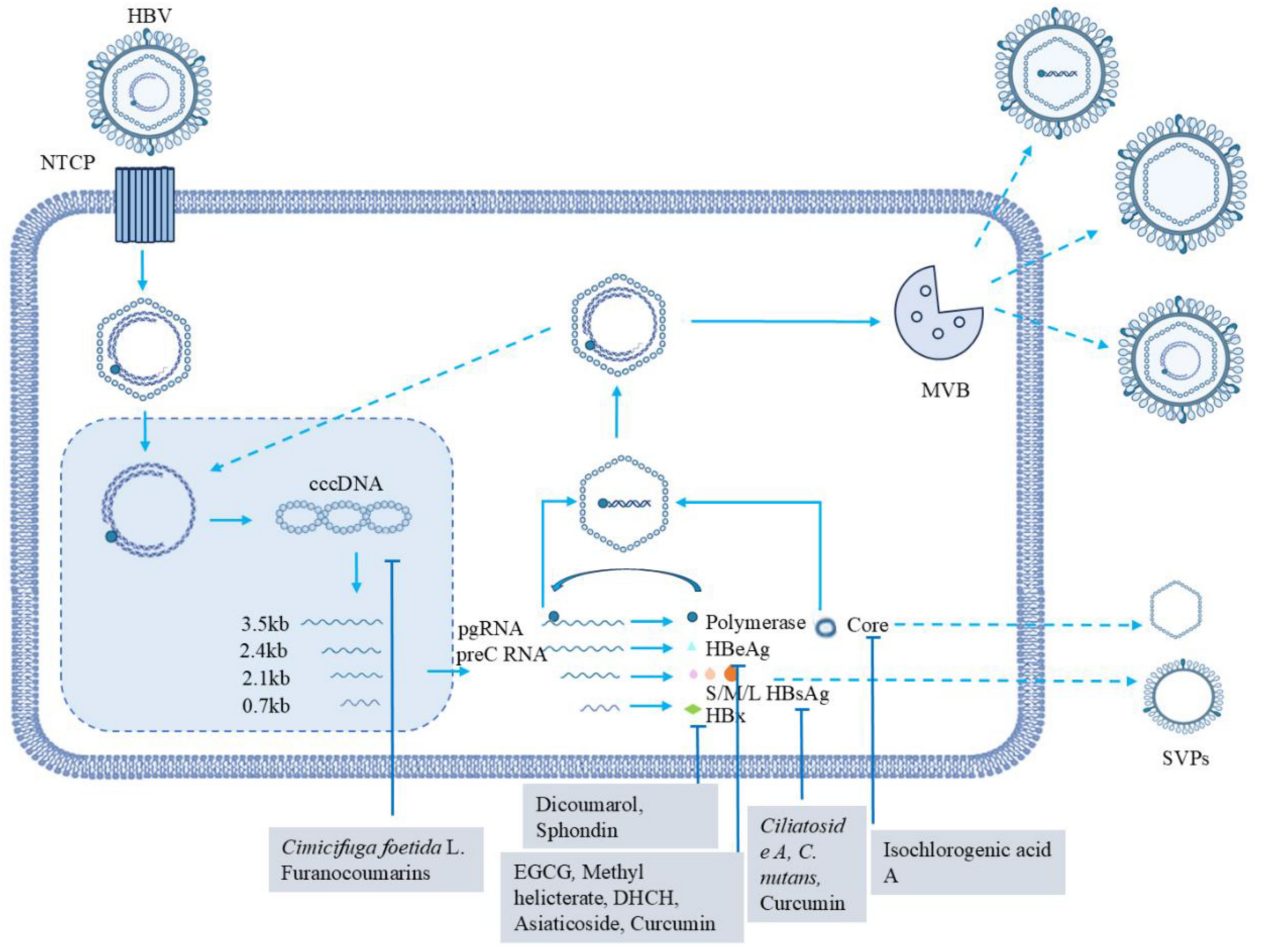
HBV life cycle and the role of natural products in modulating the process. The life cycle of HBV involves the following key steps: HBV initiates the infection process of hepatocytes by specifically binding to the NTCP receptor, which is the crucial starting event for the virus to invade host cells. Subsequently, the viral nucleocapsid is transported into the nucleus and enters the nucleus. Within the nucleus, rcDNA is converted into cccDNA under the action of host-related factors, forming a stable cccDNA molecule. Using cccDNA as a template, the transcription process is initiated, generating RNAs of different lengths (mainly 3.5kb, 2.4kb, 2.1kb, and 0.7kb). A 3.5kb RNA produces the protein product from HBcAg and polymerase; a 2.4kb RNA is translated into L-HBsAg and a 2.1kb RNA synthesizes the other two surface antigens, M-HBsAg and S-HBsAg; and a 0.7kb RNA produces HBxAg. The pgRNA interacting to HBV polymerase is selectively packaged into capsid particles. Inside the capsid particles, a DNA negative strand is synthesized using pgRNA as a template through the reverse transcription process. Subsequently, a DNA positive strand is synthesized, ultimately forming a complete viral genome. After genome replication, HBV viral particles and non-infectious SVPs are formed and secreted extracellularly. SVPs mainly refer to particles composed of HBsAg. Their self-assembled is independent of viral genome replication. For example, HBsAg can be highly expressed and self-assemble into these particles, with their secretion amount often exceeding that of infectious viral particles. Also shown in the figure are other types of secreted particles, in addition to virions and SVPs. Additionally, structures like MVBs, which may play a role in transporting. Various natural products, including *Ciliatoside* A, Dicoumarol, Sphondin, EGCG, Methyl helicterate, DHCH, Asiaticoside, Curcumin, Isochlorogenic acid A, *Cimicifuga foetida* L. and Furanocoumarins (Tyagi et al., 2024), play important inhibitory roles in the HBV life cycle.

The HBV life cycle exhibits a distinct feature where the genomic DNA, namely rcDNA, is transformed into a molecular template DNA known as cccDNA. This cccDNA serves to amplify a viral RNA intermediate, which is subsequently reverse-transcribed back to viral DNA. The remarkably high stability of cccDNA gives rise to chronic infection and leads to a relatively low cure rate. Therapeutic strategies based on natural products have been found in preclinical trials to be very promising in targeting cccDNA (**Figure 3**). We reviewed the recently published literature. Natural products play an important role in targeting cccDNA, which is significant for related research and potential therapeutic applications (**Table 1**).

**Table 1:** Natural products play an important role in targeting cccDNA.

Mechanism	Name	Source	Experimental model	Effect on cccDNA	Type	Effect	Reference
Targeting HBeAg	DHCH	*Corydalis saxicola Bunting*	HBV DNA transfected cell line HepG2.2.15	*In vitro* experiments, DHCH can inhibit the formation of intracellular HBV cccDNA in a dose-dependent and time-dependent manner. After 3-day and 6-day treatments, 25μM DHCH down-regulates the intracellular HBV cccDNA levels. The IC50​ of DHCH against intracellular HBV cccDNA was 8.25 μM	*In vitro*	Reduces HBV extracellular and cccDNA	(Zeng et al., 2013)
	Curcumin	*Curcuma longa*	Immortalized hepatocyte-like cells, HepaRG cells, HeLa, HepG2 and Huh7 cells	Treatment with 30μM curcumin reduces the level of HBV cccDNA	*In vitro*	Reduces viral load, HBeAg, HBcAg, intracellular HBV DNA, and cccDNA	(Thongsri et al., 2021)
	EGCG	Green tea	Stably expressed HBV cell line HepG2-N10	Treatment with high concentration EGCG (22.9μg/ml) down-regulates the level of cccDNA by nearly 60%. Increasing doses of EGCG resultsin progressive inhibition in the accumulation of cccDNA present within cells	*In vitro*	Reduces HBsAg and HBeAg production, extracellular HBV DNA, intracellular replication intermediates, and cccDNA	(Xu et al., 2008)
	Methyl helicterate	*Helicteres angustifolia*	*In vitro*: HepG2.2.15 cell line; *In vivo*: DHBV-infected ducklings	*In vitro*: Treatment of HepG2.2.15 cells with 15.8μM methyl helicterate for 144 h significantly reduces the HBV cccDNA content. *In vivo*: In DHBV-infected ducklings, treatment with 100mg/kg methyl helicterate significantly reduces the level of viral cccDNA in the liver compared with the infected model control ducks, and methyl helicterate reduces the liver cccDNA levels in a dose-dependent manner	*In vivo*	Decreases HBsAg/HBeAg secretion, HBV DNA, cccDNA levels	(Huang et al., 2013a)
	Asiaticoside	*Hydrocotyle sibthorpioides Lam*	*In vitro*: HepG2.2.15 cell line; *In vivo*: DHBV-infected ducklings	*In vitro*: In the HepG2.2.15 cell line, asiaticoside significantly inhibits the accumulation of cccDNA in a dose-dependent manner. After 7-day and 14-day treatments, 75μM and 150μM asiaticoside significantly reduces the cccDNA levels. *In vivo*: In DHBV-infected ducklings, asiaticoside effectively inhibits DHBV replication, reduces virus replication related to cccDNA in liver tissues, alleviated liver damage	*In vivo*	Reduces intracellular cccDNA	(Huang et al., 2013b)
Targeting HBx	Dicoumarol	Sweet clover	*In vitro*: HBV-infected HepG2-NTCP cells, HepAD38 cells, HepG2.2.15 cells, immortalized hepatocyte-like cells, etc.; *In vivo*: HBV-infected Alb-Cre transgenic mouse model, humanized liver uPA/SCID mouse model	In *in vitro* experiments, dicoumarol significantly reduces HBx expression, inhibits the transcriptional activity of cccDNA, and decreases the ratios of total RNA/cccDNA and pgRNA/cccDNA in a dose-dependent manner. In *in vivo* experiments, dicoumarol can effectively reduce the levels of serum HBsAg, HBV DNA, and intrahepatic HBV RNAs	*In vivo*	Reduces intracellular HBV RNA, DNA, and cccDNA levels	(Cheng et al., 2021)
	Sphondin	*Heracleum laciniatum*	*In vitro*: HBV-infected HepG2-NTCP cells, primary human hepatocytes, Huh-7 cells, HepAD38 cells, etc.; *In vivo*: Recombinant cccDNA mouse model (constructed by hydrodynamic injection of prcccDNA into Alb-Cre transgenic mice), humanized liver uPA/SCID mouse model	In *in vitro* experiments, sphondin binds to the Arg72 residue of the HBx protein, promotes the degradation of HBx mediated by the 26S proteasome, reduces the binding of HBx to cccDNA, reducing the levels of total HBV RNAs and 3.5-kb RNA. In *in vivo* experiments, sphondin can effectively reduce the levels of serum HBsAg, HBV DNA, as well as intrahepatic total HBV RNAs, 3.5-kb RNA, HBsAg and HBx protein	*In vivo*	Inhibits HBV cccDNA	(Ren et al., 2023)
Targeting HBsAg	Curcumin	*Curcuma longa*	HepG2.2.15 cell line	Curcumin inhibits the level of cccDNA in HepG2.2.15 cells in a time-and dose-dependent manner	*In vitro*	Inhibits HBV cccDNA	(Wei et al., 2017)
	Ciliatoside A	*Peristrophe japonica*	*In vitro*: HBV-infected HepG2-NTCP cells, primary human hepatocytes, Huh-7 cells, HepAD38 cells, HepG2.2.15 cells, etc.; *In vivo*: Recombinant cccDNA mouse model (constructed by hydrodynamic injection of prcccDNA into Alb-Cre transgenic mice)	In *in vitro* experiments, Ciliatoside A reduces HBc associated with cccDNA, and thus decreasing cccDNA transcriptional activity and reducing the production of HBV RNAs and HBsAg. In *in vivo* experiments, in the recombinant cccDNA mouse model, the Ciliatoside A treatment group significantly reduces the levels of serum HBsAg, HBV DNA, as well as intrahepatic total HBV RNAs, 3.5-kb RNA, HBV DNA, and HBsAg protein	*In vivo*	Reduces HBsAg expression and cccDNA transcriptional activity	(Fang et al., 2023)
	C. nutans	*Acanthaceae*	A mouse model of HBV infection established by hydrodynamically injecting pcDNA3.1(+)/HBV plasmid into the tail vein of male BALB/cJGpt mice	C. nutans significantly decreases the level of cccDNA in the liver tissues of mice.	*In vivo*	Reduces IL-1β, TNF-α in serum and HBV cccDNA	(Zhang et al., 2023)
Targeting HBc	Isochlorogenic acid A	Isochlorogenic acid A	HepG2.2.15 cell line	Isochlorogenic acid A significantly reduces the content of HBV cccDNA in HepG2.2.15 cells	*In vitro*	Reduces HBc stability and blocks nuclear cccDNA replenishment	(Hao et al., 2012)
cccDNA inhibition	*Cimicifuga foetida* L.	Mainly composed of C. foetida, Kudzuvine root, Chinese herbaceous peony and liquorice	60 patients with CHB divided into two groups. Group I received adefovir, and group II received a combination therapy of adefovir and C. foetida for over 48 weeks	In patients with CHB, *Cimicifuga foetida* L., when combined with adefovir, significantly reduces the median HBV cccDNA level	Clinical study	Reduces median cccDNA	(Dai et al., 2016)
	Furanocoumarins Fc-20 and Fc-31	Plant	HBV-infected HepG2-NTCP cells and HepG2.2.15 cells	50 μM Fc-20/Fc-31 reduce cccDNA in a concentration-dependent manner	*In vitro*	Fc-20/Fc-31 induce HBx proteasomal degradation and decrease H3K4me3 on cccDNA to suppress transcription	(Tyagi et al., 2024)

EGCG, epigallocatechin gallate; DHCH, Dehydrocheilanthifolin; *C. nutans*, *Clinacanthus nutans* (Burm.f.) Lindau, IL-1β: interleukin-1β.

### 5.1 Targeting HBeAg

#### 5.1.1 Research background of HBeAg

HBeAg is a non-structural secreted protein, although its expression is not essential for maintaining infection. This antigen is clinically used as an indicator of viral replication, infectivity, disease severity, and treatment response (Milich and Liang, 2003).

#### 5.1.2 Natural products targeting HBeAg

##### 5.1.2.1 In vitro

###### 5.1.2.1.1 Corydalis saxicola-dehydrocheilanthifolin

*Corydalis saxicola*, a traditional Chinese medicine, has been used to treat various liver diseases. Total alkaloids containing *Corydalis saxicola* show effectiveness against hepatitis B, liver fibrosis, and NAFLD (Li et al., 2008; Wang et al., 2022; Wu et al., 2022; Guo et al., 2024). Dehydrocheilanthifolin (DHCH), a quaternary ammonium alkaloid isolated from *Corydalis saxicola*, effectively inhibits HBsAg and HBeAg secretion in HepG2.2.15 cells (Zeng et al., 2013). DHCH reduces HBV extracellular and intracellular DNA while increasing pgRNA levels, potentially due to the reduction of cytoplasmic mature virions and cccDNA (Zeng et al., 2013). Three possibilities may explain the interference of DHCH with the intracellular replication cycle of HBV. First, DHCH may disrupt the packaging of pgRNA into the nucleocapsid, leading to the accumulation of pgRNA, which in turn results in a reduction of viral DNA and nuclear cccDNA. Second, the recycling of the viral genome from immature virus particles to the cccDNA pool may be disrupted by DHCH. Third, DHCH may inhibit the activity of viral DNA polymerase, which also leads to a reduction of rcDNA and cccDNA and an accumulation of pgRNA. Consequently, the decrease in the cccDNA pool triggered by DHCH might be ascribed to the diminished cytoplasmic mature virions instead of the direct inhibition of the conversion from rcDNA to cccDNA. Further research demonstrated that DHCH could reduce the levels of extracellular DNA, intracellular DNA, and cccDNA of HBV in a manner that depends on both dose and time. The IC50 values were determined to be 15.08 μM, 7.62 μM, and 8.25 μM respectively.

###### 5.1.2.1.2 Turmeric-curcumin

Curcumin, a polyphenol derived from turmeric (*Curcuma longa*) (Wan Mohd Tajuddin et al., 2019), reduces viral load, HBeAg, HBcAg, intracellular HBV DNA, and cccDNA levels in infected-differentiated immortalized hepatocyte-like cells (d-imHCs) cells (Thongsri et al., 2021). During the early and replication stages of the HBV life cycle, curcumin treatment can reduce intracellular HBV DNA, viral load, and HBeAg. The HBV cccDNA level was lessened by a half after 30µM curcumin.

###### 5.1.2.1.3 Green tea-epigallocatechin gallate

Green tea (*Camellia sinensis*, Theaceae) is one of the most popular beverages globally, especially in Asia (Forester and Lambert, 2011). Subacute toxicity studies indicate that daily consumption of green tea is safe with no adverse effects in mice (Hsu et al., 2011). Human liver cancer cell line HepG2-N10 was utilized. The cell line was created through the transfection of HepG2 cells with a transfer plasmid containing a 1.3 unit length of genotype A HBV genome (subtype adw2). Epigallocatechin gallate (EGCG) was investigated for its anti-HBV effects. EGCG interferes with viral gene transcription to reduce HBsAg and HBeAg production. It also affects viral protein processing or transport, impeding antigen secretion. At the DNA replication stage, it can inhibit viral DNA polymerase, reducing extracellular HBV DNA and intracellular replication intermediates. For cccDNA, it disrupts related mechanisms, like interfering with proteins bound to cccDNA, to affect its function. EGCG reduces HBsAg and HBeAg production, intracellular replication intermediates, and cccDNA levels in a dose-dependent manner (Xu et al., 2008). Upon treatment with a high concentration of EGCG (22.9μg/ml), the level of cccDNA decreased by approximately 60%.

##### 5.1.2.2 In vivo

###### 5.1.2.2.1 Helicteres angustifolia-methyl helicterate

*Helicteres angustifolia* (Sterculiaceae) is traditionally used for treating immune disorders and liver diseases (Zhang et al., 2018). Methyl helicterate, a triterpenoid isolated from *Helicteres angustifolia* (Sterculiaceae) (Huang et al., 2012), significantly decreases HBsAg, HBeAg secretion, HBV DNA, cccDNA levels, and viral RNA in HBV-transfected HepG2.2.15 cells without affecting mitochondrial DNA content. In this study, a significant reduction in intracellular cccDNA levels was observed after treatment with 15.8μM. In DHBV-infected ducklings, methyl helicterate reduces serum DHBV DNA, liver total viral DNA, and cccDNA levels, suggesting its efficacy in inhibiting HBV replication (Huang et al., 2013a).

###### 5.1.2.2.2 Hydrocotyle sibthorpioides-asiaticoside

*Hydrocotyle sibthorpioides* (Apiaceae *Hydrocotyle sibthorpioides* Lam.) has been used in folk medicine to treat HBV infection, fever, edema, and sore throat (Huang et al., 2013b). This study evaluated the anti-HBV activity of saponins by detecting the levels of HBV antigens, extracellular HBV DNA, cccDNA, and HBV promoters in HepG2.2.15 cells. Treatment with 75 μM and 150 μM asiaticoside for 7 and 14 days resulted in decreased cccDNA levels compared to the control. Asiaticoside, an active compound, significantly inhibits HBsAg, HBeAg, extracellular HBV DNA, and intracellular cccDNA levels in HepG2.2.15 cells. It also reduces viral DNA transcription and replication by inhibiting CP, SP1, SP2, and XP activity (Huang et al., 2013b). In addition, the levels of serum HBsAg, HBeAg, Duck Hepatitis B Virus (DHBV) DNA are analyzed in DHBV-infected ducks. Asiaticoside also significantly reduces HBsAg, HBeAg and DHBV DNA, alleviating liver injury and liver pathology (Huang et al., 2013b).

### 5.2 Targeting HBx

#### 5.2.1 Role of HBx in HBV replication

During the virus’s life cycle, the HBx protein plays a crucial role in initiating and maintaining HBV replication (Lucifora et al., 2011). HBx also upregulates the degradation of apolipoprotein B mRNA editing catalytic polypeptide-like (APOBEC)3B, thereby increasing cccDNA (Gao et al., 2017). Therapeutic trials based on HBx are only at the preclinical stage.

#### 5.2.2 Natural products targeting HBx

##### 5.2.2.1 In vivo

###### 5.2.2.1.1 Sweet clover-dicoumarol

Dicoumarol, a coumarin−like compound derived from sweet clover [*Melilotus officinalis* (L.) Pall], has various pharmacological activities, including anticoagulant, antitumor, and antibacterial effects (Ge et al., 2024). NAD(P)H quinone dehydrogenase 1 (NQO1) knockout or dicoumarol (an inhibitor of NQO1) treatment significantly reduces HBx-mediated cccDNA recruitment and inhibits cccDNA transcriptional activity (Cheng et al., 2021). Dicoumarol dose-dependently reduces HiBiT-HBx levels with an EC50 of 100μM in HepG2 cells. It shortens the half-life of HBx protein without affecting the stability of other viral proteins (e.g., HBs, HBc, and viral polymerase). In HBV-infected cells, dicoumarol decreases total HBV RNAs, pgRNA, HBsAg, HBc, and HBx protein levels, and reduces the ratios of total RNAs/cccDNA and pgRNA/cccDNA, indicating inhibition of cccDNA transcription without affecting cccDNA levels. In Alb-Cre transgenic mice, it effectively reduces serum HBsAg, hepatic HBV RNAs, HBx and HBsAg levels, decreases HBx-cccDNA binding, and alters histone modifications on cccDNA. In human liver-chimeric uPA/SCID mice, dicoumarol treatment reduces serum HBsAg and HBV DNA, hepatic HBV DNA and RNAs, and HBx levels associated with cccDNA. NQO1 stabilizes HBx protein by inhibiting 20S proteasome-mediated HBx degradation, thereby increasing HBx expression and half-life. The promotion of cccDNA transcription by NQO1 depends on HBx. NQO1 stabilizes HBx, promotes HBx binding to cccDNA. As an NQO1 inhibitor, dicoumarol promotes HBx degradation, reduces HBx binding to cccDNA, and thereby inhibiting cccDNA transcription.

###### 5.2.2.1.2 Heracleum laciniatum-sphondin

Sphondin, a furanocoumarin derivative isolated from *Heracleum laciniatum*, inhibits both intracellular HBsAg production and HBV RNAs levels. Sphondin also binds preferentially to the HBx protein via residue Arg72, leading to increased HBx degradation through the 26S proteasome (Ren et al., 2023). Sphondin significantly reduces the recruitment of HBx to cccDNA, inhibiting cccDNA transcription and HBsAg expression (Ren et al., 2023). The absence of HBx or R72A mutations eliminates the anti-viral effects of sphondin in HBV-infected HepG2-NTCP cells and primary human hepatocytes cells (Ren et al., 2023).

### 5.3 Targeting HBsAg

#### 5.3.1 Significance of HBsAg in HBV diagnosis

HBsAg is an important diagnostic marker for HBV infection.

#### 5.3.2 Natural products targeting HbsAg

##### 5.3.2.1 In vitro

###### 5.3.2.1.1 Turmeric-curcumin

In addition, other studies have also shown that curcumin has an anti-HBsAg effect. In the anti-HBV study using HepG2.2.15 cells stably transfected with HBV, 20μM curcumin with histone deacetylase inhibitors were applied. Curcumin significantly decreases intracellular HBV cccDNA, replication intermediates and mRNA. Upon treatment of HepG2.2.15 cells with 20μM curcumin for two days, the levels of HBsAg and cccDNA exhibited significant reductions of 57.7% and 75.5%, respectively. This indicates that curcumin effectively suppresses HBV antigen expression and cccDNA levels in a dose- and time-dependent manner, suggesting its potential as a therapeutic agent against HBV infection. Curcumin reduces histone H3/H4 acetylation and that of cccDNA-binding histones. The inhibitor blocks its HBV-inhibiting effect, indicating it acts via histone deacetylation. Combined with HBx/HBs-siRNAs, it enhances HBV inhibition, further reducing HBsAg and replication intermediates (Wei et al., 2017).

##### 5.3.2.2 In vivo

###### 5.3.2.2.1 Peristrophe japonica-ciliatoside A

*Peristrophe japonica*, traditionally used for antibacterial, anti-inflammatory, and cough relief, has demonstrated a strong inhibitory effect on HBsAg secretion (Fang et al., 2023). Ciliatoside A, a monomer compound extracted from *Peristrophe japonica*, exhibits potent anti-HBV effects in HBV-infected cells and HBV recombinant cccDNA mice model (Fang et al., 2023). It significantly reduces HBsAg expression and cccDNA transcriptional activity without toxicity. The mechanism involves the induction of autophagy via the AMPK/ULK1/mTOR axis, leading to inhibited HBV transcription and replication (Fang et al., 2023).

###### 5.3.2.2.2 Clinacanthus nutans

*Clinacanthus nutans* (Burm.f.) Lindau (*C. nutans*), a member of the Acanthaceae family, is used for treating skin infections, insect bites, microbial infections, and cancer. Its extracts possess antiviral, anticancer, and antioxidant properties (Chia et al., 2021). Research has shown that *C. nutans* exerts protective efects in HBV model mice. *C. nutans* disrupts HBsAg production, reduces interleukin-1β (IL-1β), TNF-α in serum and HBV cccDNA in HBV model mice (Zhang et al., 2023).

### 5.4 Targeting HBc

#### 5.4.1 Function of HBc in cccDNA regulation

HBc, commonly referred to as a component of the HBV capsid, plays also roles in the stability, transcription, and epigenetic regulation of cccDNA (Van Damme et al., 2021).

#### 5.4.2 Natural product targeting HBc

##### 5.4.2.1 In vitro

###### 5.4.2.1.1 Medicinal plants-isochlorogenic acid A

Studies have shown that natural products can target HBc to inhibit HBV. Isochlorogenic acid A, a dicaffeoylquinic acid found in various medicinal plants and vegetables (Tang et al., 2023), has anti-inflammatory, hepatoprotective, and antiviral properties (Liu et al., 2020). The anti-HBV target of isochlorogenic acid A may be related to blocking the translation step of HBV replication. Isochlorogenic acid A significantly decreased the content of cccDNA and markedly induced the expression of heme oxygenase-1 (HO-1) in HepG2.2.15 cells. The overexpression of HO-1 may promote the anti-HBV activity of isochlorogenic acid A by reducing the stability of the HBc protein, thereby blocking the refilling of nuclear HBV cccDNA (Hao et al., 2012). The hepatoprotective effects of isochlorogenic acid A may also be linked to its antioxidant activity and HO-1 induction (Hao et al., 2012).

### 5.5 Inhibition of cccDNA

#### 5.5.1 Research rationale for cccDNA inhibition

Currently, research is underway to inhibit the transcription/replication of cccDNA, which will reduce the antigen load of HBV.

#### 5.5.2 *Cimicifuga foetida* L. in combination therapy

##### 5.5.2.1 Clinical study

*Cimicifuga foetida* L., a traditional Chinese medicine primarily composed of *Cimicifuga foetida L*. Kudzuvine root, is used for its anti-inflammatory, antipyretic, and analgesic effects (Gai et al., 2012). Combined therapy with *Cimicifuga foetida* L. and adefovir significantly reduces median cccDNA levels, serum transforming growth factor-β (TGF-β), and interferon-γ (IFN-γ) levels in patients (Dai et al., 2016). The cccDNA, HBsAg, HBeAg and serum HBV DNA are reduced in the patients with CHB after combined therapy with *Cimicifuga foetida* L. (Tang et al., 2023). Although adefovir can rapidly turn HBV DNA negative, it has limited effect on cccDNA. If the drug is stopped at this time, there is often a virological rebound and even a worsening of the disease. Moreover, cccDNA can still be detected in the liver even after receiving anti-viral therapy for more than 10 years (Tajiri and Shimizu, 2016; Chauhan et al., 2018).

##### 5.5.2.2 Plant-furanocoumarins

Furanocoumarins are naturally occurring compounds in the plant world (Sumorek-Wiadro et al., 2024). Furanocoumarins Fc-20 and Fc-31 can reduce the level of cccDNA and 3.5 kb RNA transcription in HBV-infected HepG2-NTCP and HepG2.2.15 cells. The mechanism is to induce the degradation of HBx protein through the proteasome, reduce its binding to cccDNA and lower the active marker of H3K4me3. The combination with entecavir can enhance the inhibition of 3.5 kb RNA and HBV DNA, but has no superposition effect on cccDNA (Tyagi et al., 2024).

In conclusion, although most natural products targeting cccDNA are still at the *in vitro* or preclinical *in vivo* stage, *Cimicifuga foetida* L. is the only compound with published clinical evidence, as shown in a small-scale study (n=60) (clinical stage). The combined use with Adefovir can reduce the median cccDNA level in patients with chronic hepatitis B. The other natural products mentioned above have not yet entered human trials, but methyl helicterate and asiaticoside have shown hope in preclinical animal models (such as DHBV-infected duckings), and further toxicological studies and formulation optimizations can be prioritized to advance clinical trials. Compounds like curcumin, despite their strong *in vitro* activity, are hindered due to poor bioavailability and the lack of continuous clinical development for HBV.

However, the majority of studies focus on the standalone effects of natural products, with only a few cases (such as *Cimicifuga foetida* L. and furanocoumarins) exploring their combined application with antiviral drugs. Future research should place greater emphasis on the collaborative use of natural products and antiviral agents. It is worth noting that although all these studies have reported the anti-HBV and inhibitory effects on cccDNA of these natural products. However, the pharmacological characteristics of these natural products (such as ADMET properties: absorption, distribution, metabolism, excretion, toxicity) are currently unclear. These data are crucial for evaluating drug-drug interactions, optimal dosages and long-term safety, especially considering the complexity of multiple herbal formulations. These data are crucial for assessing its potential for clinical transformation and should be addressed in future studies.

## 6 Clinical challenges and limitations

Natural products encounter significant pharmacokinetic restrictions during their journey towards clinical application for HBV treatment. The most pressing issue is the poor pharmacokinetic properties of natural products, primarily characterized by low solubility and rapid metabolism. For example, curcumin, despite its potent anti-HBV activity in *in vitro* and some animal models, has limited therapeutic potential due to its hydrophobic nature leading to low oral bioavailability. To overcome this, the development and application of advanced formulation technologies, such as nanocarriers, should be prioritized. Nanocarriers can encapsulate hydrophobic natural products, enhancing their solubility and enabling targeted delivery to liver cells, thereby improving bioavailability.​

Herb-drug interactions also pose significant risks. Given that natural products contain complex mixtures of bioactive compounds that can interact unpredictably with other medications, rigorous clinical trials and in-depth pharmacokinetic studies are essential. These studies should aim to thoroughly understand the interaction mechanisms and establish clear guidelines for the safe co-administration of natural products and conventional drugs.​

The differences between entire extracts and separated substances represent another key challenge. Whole extracts feature multiple components with potential synergistic or antagonistic interactions that affect the overall therapeutic effect, while separated substances may lack certain beneficial properties. Comprehensive comparative studies, integrating chemical analysis with biological activity assays, are needed to determine the most effective form for treatment.​

Batch variability of plant extracts is a major obstacle to standardization and large-scale production. The active components in natural extracts vary greatly depending on factors like the plant’s growth environment, harvest time, extraction methods, and storage conditions. To tackle this, strict quality control measures must be implemented. This includes standardizing the growth and harvest of plant materials, optimizing extraction processes, and establishing comprehensive quality evaluation systems using techniques such as high-performance liquid chromatography (HPLC) and mass spectrometry (MS). By concentrating on these priority areas and advancing along the proposed research directions, substantial progress can be achieved in the clinical utilization of natural products for HBV treatment.

## 7 Conclusions and prospects

Natural products have emerged as a potential avenue in the fight against HBV infection, particularly in terms of their ability to target cccDNA. HBV has a complex structure. Its virion consists of an outer envelope containing HBsAg. The L protein domain preS1, which binds to NTCP, plays a role in virus entry. It triggers the host immune response and is a major target for diagnostic and therapeutic interventions. The immune response is triggered mainly against the SVPs which are secreted in a high ratio compared to the virions. The functional cure for HBV is defined as undetectable HBsAg, besides undetectable viraemia (Lee and Ahn, 2011). The core of the virus houses the viral genome and is formed by HBc which self-assemble into an icosahedral capsid. Additionally, HBeAg, a secreted protein, is related to viral replication and serves as an indicator of active infection and virus spread (Kao, 2008). The cccDNA, a key reservoir for HBV within hepatocytes, is crucial for the virus’s persistence (Nevola et al., 2023). Several natural compounds, sourced from traditional Thai and Chinese medicines, have demonstrated the ability to reduce cccDNA levels. This reduction is likely associated with their inhibitory effects on viral replication, which may be due to interference with the functions of viral proteins. For instance, the suppression of HBsAg may result from the modulation of viral gene expression or the disruption of viral protein synthesis and trafficking. By targeting cccDNA and related viral processes, these natural products offer the potential for developing novel antiviral therapies to achieve a functional cure for HBV. The following will elaborate on the existing research gaps in several key aspects.

Active component identification and mechanism elucidation: Regarding the application of natural products in anti-HBV research, the relationship between their structures and effects are a crucial aspect. In particular, many natural products are mixtures, which makes the situation even more complicated. Taking the extract of a certain traditional Chinese medicine as an example, it may contain multiple chemical components, such as flavonoids, terpenoids, alkaloids, and so on. These compounds of different structural types may each act on different targets of HBV or enhance the antiviral effect through mutual synergy. For example, flavonoid compounds may inhibit the activity of viral proteins by binding to them, terpenoid compounds may affect the process of viral cell membrane fusion, and alkaloid compounds may interfere with viral gene transcription. However, due to the complexity of the mixtures, accurately identifying the active components and analyzing their structures and action mechanisms face huge challenges. In current research, advanced separation and purification technologies, such as HPLC and MS, need to be used to finely separate and identify the structures of natural product mixtures (Hughes, 2021). Meanwhile, combined with bioactivity tests, the antiviral activities of each component are determined, and then their action mechanisms are further studied.

Efficacy variability and stability: Although natural products have achieved certain results in anti-HBV research, they still face many challenges on the road to clinical application (Atanasov et al., 2021). The efficacy of natural products varies significantly. Taking the traditional solvent extraction method as an example, slight changes in parameters such as the types, concentrations, extraction times, and temperatures of extraction solvents may have a significant impact on the active components in the final extract. In addition, the stability of natural products is also a key factor. Many natural products are prone to undergo chemical reactions such as degradation, oxidation, or isomerization under different environmental conditions, such as light, temperature, and humidity, thereby losing their antiviral activities.

Bioavailability and molecular mechanisms: Poor bioavailability is a common issue with natural products. Many natural compounds have low solubility and are rapidly metabolized, limiting their effectiveness. For example, curcumin exhibits strong anti-HBV activity cell experiments and some animal models. However, due to its poor water solubility, it is difficult to be effectively absorbed by the gastrointestinal tract after oral administration (Mohanty and Sahoo, 2017). The specific molecular mechanism by which natural products act on cccDNA has not been fully elucidated. Although some signaling pathways have been discovered in current research, the details of the precise molecular interactions between these pathways and natural products still need to be further explored. Natural products can interact with multiple intracellular molecules.

Cytotoxicity and quality control: Some natural products may exhibit cytotoxicity at high concentrations. Determining the balance between their anti-HBV activity and cytotoxicity is essential. For natural products that exhibit cytotoxicity at high concentrations, such as *C. nutans*, the balance between their anti-HBV activity and cytotoxicity needs to be further studied. Such a system includes the use of multiple cell models, a comprehensive assessment of different detection indicators (such as cell viability, apoptosis rate, oxidative stress level, etc.), and a thorough study of the pharmacokinetics and toxicology of drugs in animal models. Natural extracts typically contain multiple active components. The types, contents, and proportions of these components often vary significantly among different source materials and extraction batches. This complexity makes the standardization and quality control of natural products a thorny problem. In terms of quality control, due to the complexity of the components, conventional chemical analysis methods are difficult to comprehensively and accurately detect and monitor the quality of natural products.

Despite the numerous challenges mentioned above, it is encouraging that several classes of highly promising compounds have been identified from a wide range of natural products in existing research. These compounds stand out in terms of their anti-HBV activity, especially in their effects on cccDNA, pointing the way for future research and drug development. The following will provide a detailed introduction to these potentially valuable classes of compounds. Promising Classes of Compounds: Flavonoid-rich compounds like EGCG from green tea show anti-HBV potential, with high-concentration EGCG (22.9μg/ml) reducing cccDNA levels by nearly 60%. Terpenoid-based compounds such as methyl helicterate from Helicteres angustifolia can decrease HBV cccDNA content *in vitro* and in DHBV-infected ducklings. Alkaloid-containing compounds like DHCH from Corydalis saxicola Bunting can inhibit intracellular HBV cccDNA formation in a dose - and time - dependent manner. These classes of compounds hold promise for anti-HBV drug development.

Future research directions include enhancing the pharmacokinetic properties of natural products. To enhance the pharmacokinetic properties of natural products, we can utilize nanocarrier technology or chemical modification methods. Nanocarriers can encapsulate or adsorb natural products on their surfaces to achieve targeted drug delivery (Solís-Cruz et al., 2021). For example, by designing nanocarriers that specifically target liver cells, the enrichment concentration of natural products in the liver can be increased, their distribution in other tissues can be reduced, thereby reducing potential toxicity and improving antiviral efficacy. Meanwhile, nanocarriers can also protect natural products from the influence of the internal environment of the body and improve their stability (Bennani et al., 2024). Chemical modification can modify the molecular structures of natural products (Ding and Xue, 2024), such as introducing water-soluble groups to improve their solubility or modifying functional groups to enhance their affinity for targets, thereby improving their bioavailability and activity. In-depth exploration of molecular mechanisms should integrate multi-disciplinary methods like genomics, proteomics, and systems biology to study viral genome expression, protein changes, and construct models for predicting drug effects and risks, identifying key targets and biomarkers for personalized medicine. Developing combination therapies by using natural products in conjunction with existing antiviral therapies (such as nucleotide analogues) or other natural compounds can inhibit multiple viral life-cycle stages through different mechanisms, improving efficacy and reducing drug resistance. Additionally, optimizing drug delivery systems for precise liver-targeting and establishing a more comprehensive quality control system with advanced analysis techniques can reduce adverse effects on other tissues and ensure product quality stability, accelerating the translation of natural products from laboratory to clinical applications.

In addition to natural products, emerging non-natural strategies targeting cccDNA are rapidly advancing, offering novel avenues for the complete cure of chronic hepatitis B. The CRISPR-Cas9 system, guided by gRNA, cleaves cccDNA to induce double-strand breaks, leading to sequence mutations or degradation via non-homologous end joining (NHEJ) (Ma et al., 2014). For example, hydrodynamic injection of CRISPR-Cas9 expression vectors enhances cccDNA clearance, though off-target effects and chromosomal translocation risks require attention. Base editing technologies (e.g., cytosine base editors, CBEs) introduce precise point mutations (e.g., C>T) in cccDNA without double-strand breaks, effectively silencing viral gene expression and reducing HBsAg secretion with higher safety profiles (Smekalova et al., 2024). Non-viral delivery systems, such as lipid-like nanoparticles (LLNs) and near-infrared-responsive biomimetic nanoparticles (UCNPs-Cas9@CM), efficiently deliver Cas9/gRNA to the liver, minimizing immunogenicity and off-target rates (Jiang et al., 2017; Wang et al., 2022). Furthermore, small-molecule compounds like disubstituted sulfonamides (DSS) inhibit *de novo* cccDNA synthesis, while cccDNA reducers (e.g., ccc_R08) selectively reduce cccDNA levels without significant cytotoxicity (Wang et al., 2023). In epigenetic regulation, CRISPR interference (CRISPRi) fuses with KRAB domains to promote heterochromatin formation and silence cccDNA transcription. For instance, siRNA or small molecules targeting HBx (e.g., nitazoxanide) disrupt the HBx-DDB1 complex, restoring SMC5/6-mediated inhibition of cccDNA (Song et al., 2021). These non-natural strategies complement natural products, providing diversified technical pathways to overcome the therapeutic challenge of HBV cccDNA, though further optimization is needed for delivery efficiency, long-term safety, and clinical translation.

In conclusion, natural products have broad application prospects in targeting cccDNA for HBV treatment, but their limitations must be carefully considered before clinical application. Continuous research and development efforts are indispensable to overcome these challenges.
